# Interferometric SAR Phase Denoising Using Proximity-Based K-SVD Technique

**DOI:** 10.3390/s19122684

**Published:** 2019-06-14

**Authors:** Chandrakanta Ojha, Adele Fusco, Innocenzo M. Pinto

**Affiliations:** 1 School of Earth and Space Exploration, Arizona State University, Tempe, AZ 85281, USA; cojha1@asu.edu; 2 CNR IREA, Via Diocleziano 328, 80124 Naples, Italy; 3 Università del Sannio, Palazzo Dell’Aquila Bosco Lucarelli, Corso Garibaldi, 107 82100 Benevento, Italy; pinto@sa.infn.it; 4 National Institute for Nuclear Physics, Department of Naples, Strada Comunale Cinthia, 80126 Naples, Italy

**Keywords:** SAR (Synthetic Aperture Radar) interferograms, dictionary learning, sparse representation, ProK-SVD, proximity operator, *l*^0^-minimization, simulated data

## Abstract

This paper addresses the problem of interferometric noise reduction in Synthetic Aperture Radar (SAR) interferometry based on sparse and redundant representations over a trained dictionary. The idea is to use a *Proximity-based K-SVD (ProK-SVD)* algorithm on interferometric data for obtaining a suitable dictionary, in order to extract the phase image content effectively. We implemented this strategy on both simulated as well as real interferometric data for the validation of our approach. For synthetic data, three different training dictionaries have been compared, namely, a dictionary extracted from the data, a dictionary obtained by a uniform random distribution in [−π,π], and a dictionary built from discrete cosine transform. Further, a similar strategy plan has been applied to real interferograms. We used interferometric data of various SAR sensors, including low resolution C-band ERS/ENVISAT, medium L-band ALOS, and high resolution X-band COSMO-SkyMed, all over an area of Mt. Etna, Italy. Both on simulated and real interferometric phase images, the proposed approach shows significant noise reduction within the fringe pattern, without any considerable loss of useful information.

## 1. Introduction

Interferometric Synthetic Aperture Radar (InSAR) [1,2,3,4,5,6,7,8,9] is a consolidated remote sensing technique with broad applications in the field of Earth and environmental sciences. In the last two decades, InSAR has been playing a significant role for measuring several geophysical quantities, including land topography, surface deformations, land changes, water levels, ocean currents, soil moisture, glacier dynamics and vegetation properties [10]. Basically, InSAR uses two coherent SAR images to form an interferogram that can be acquired either from two different antennas on the same platform or from different passes of the same antenna at different times. In general, SAR interferograms are affected by several decorrelation effects, depending on different noise sources, which collectively produce interferogram phase noise. Decorrelation stems from system noise, processing errors and other internal and external factors (e.g., atmospheric fluctuations) [6,9,11]. In the last decade, several techniques have been proposed in the literature for getting rid of the decorrelation effects in the interferometric phase noise. Non-adaptive filtering methods, including the mean filtering technique proposed by Rosen [9], are not so effective for InSAR interferograms. In fact the adoption of some fixed windows for filtering can induce phase distortions due to the periodic character of interferograms not being considered. Another well known filtering method is the adaptive noise filtering proposed by Lee [12]. This method uses suitably selected windows, whose orientations better fit the fringes. Although it has an advantage over the previous non-adaptive filtering methods, it uses phase unwrapping *before* filtering, and phase rewrapping after, resulting in potentially poor accuracy, and slow processing. Goldstein and Werner proposed a frequency domain adaptive filter algorithm [7] that presents the limitation that for high values of filter parameter α, a residual systematic phase trend appears, indicating a loss of resolution in the filtered phase. Baran et al. [13] proposed a modification of Goldstein filter that makes the parameter of the filter dependent on the interferogram coherence. In recent years, Feng et al. [14] suggested a further modification of Goldstein filter in order to preserve fringe edges. Suo et al. [15] designed a strategy that makes use of a coherence-adaptive window size to suppress the phase noise, compensating the correlation effects induced by the terrain topography.

An interferogram typically exhibits structures on different scales due to varying fringe density. Hence multiresolution techniques could be appropriate tools for SAR interferogram denoising. Accordingly, López-Martínez and Fàbregas introduced a noise reduction algorithm in the complex wavelet domain [16], further elaborated in [17]; Suksmono and Hirose [18] used a fifth order complex-valued Markov random-field model and a residue-based adaptive multiresolution technique; a non-local multiresolution method has been proposed in [19,20].

Recently, a new emerging technique named Compressive Sensing (CS) [21] has been extensively applied in many applications of optical image processing. Unitary wavelet coefficients, leading to shrinkage algorithm [22,23,24,25,26] are firstly used, and then, because of regular separable 1-D wavelets are not well suited for handling images, curvelet [27], contourlet [28], wedgelet [29], bandlet [30], and the steerable wavelet [31] were investigated. Further, introduction of Matching Pursuit (MP) [32] and Basis Pursuit (BP) denoising [33] allowed to address the image denoising problem as a direct sparse decomposition problem over redundant dictionaries [34]. The CS technique has also been used in SAR imaging [35,36,37]. Several applications to object detection in SAR images are discussed in [38]. Yet, very few algorithms for InSAR denoising have been proposed, based on CS [39,40]. In this context, we address the interferometric phase image denoising problem by solving the related noise-free phase estimation problem, using an efficient technique, namely the K-Means Singular Values Decomposition (henceforth K-SVD [34]), capitalizing on sparse representation over trained dictionary [41]. In addition we introduce a proximity concept in applying K-SVD to image-patches, that generally improves its performance at almost no cost.

When approaching a general inverse problem in image processing using the Bayesian approach, an image prior is needed (spatial smoothness, low/max-entropy, or sparsity in some transform domain). In our case, the prior is represented by the observation that the original phase image without noise should be smooth within the fringes. The idea is trying to extract this data structure directly from images; this corresponds to learning the dictionary from data themselves [30,42,43,44]. In this work, we compare three options for initial dictionary selection: (1) building the dictionary using the discrete cosine transform, (2) building the dictionary using patches on simulated data corrupted by additive noise, and real InSAR phase images from different platforms for training, and (3) building a random dictionary. The K-SVD algorithm [45,46] merges together training and denoising into one coherent and iterative process [34]. The approach is based on handling small image patches [34,47] rearranged in a 1D array using a proximity concept in order to preserve the spatial correlation among adjacent pixels. In particular in [47] this local approach has been used for turning a local Markov Random Field-based prior into a global one. Following the same strategy, we obtain global and efficient denoising by using a global image prior that forces sparsity over patches at every point of the image (with overlaps) [48,49,50]. This paper is accordingly organized as follows. Section 2 provides a general formulation of the denoising problem in the context of SAR interferometric data, and describes the rationale of using ProK-SVD approach, as well its practical implementation. Numerical experiments and discussion are presented in Section 3; summary and conclusions follow in Section 4.

## 2. The Proximity-Based K-SVD Methods for SAR Interferogram Denoising

This section introduces the theoretical framework of a *Proximity-based K-SVD (ProK-SVD)* algorithm for denoising SAR interferometric data, relying on dictionary learning, sparse representations, and clever proximity-driven rearrangement of the data.

### 2.1. Fundamental of Denoising Problem in Interferometry

To set the stage, let us start by considering one single SAR interferogram *I*, obtained from two co-registered SAR acquisitions $S_{1}$ and $S_{2}$ in InSAR data processing. Let $\phi_{1}$ and $\phi_{2}$ represent the phase information of the acquired images $S_{1}$ and $S_{2}$, such that:

(1) $$I=S_{1}\;\cdot \;S_{2}^{*}=\left|S_{1}\right|\left|S_{2}\right|e^{j(\phi_{1}-\phi_{2})}=\left|S_{1}\right|\left|S_{2}\right|e^{j\phi_{m}}$$

where $\phi_{m}=\phi_{1}-\phi_{2}$ represents the interferometric phase [8,51]. In general, measurements of the interferometric phase are both noisy (due to various decorrelation effects such as sensor noise, temporal and geometrical fluctuations), and affected by undeterminate additive multiples of 2π (periodic functions, *wrapped phase*). Consequently retrieving the noise free $\phi_{m}$ from noisy data is a very difficult inverse problem [40]. Given a phase ϕ∈ℜ, the corresponding *wrapped* interferometric phase $\phi_{2\pi }$ can be expressed as

(2) $$\begin{array}{c} \phi_{2\pi }=W\left(\phi \right)\\ W:\phi \in ℜ\to \phi_{2\pi }\in \left.\left[-\pi ,\pi \right.\right) \end{array}$$

where

(3) $$\phi_{2\pi }=\left(\phi \;\mathrm{mod}\;2\pi \right)-\pi$$

and (amodb) is the remainder of a/b. The main goal of the interferometric phase estimation problem is to figure out the 2D phase map $\phi_{2\pi }$ from the observed 2D map $\phi_{z}$ given by

(4) $$\phi_{z}=\phi_{2\pi }+\nu$$

where ν represents noise [1,2,3,4,5,6,7,8,9,52]. Our aim is evaluating an estimate ${\hat{\phi }}_{2\pi }$ of $\phi_{2\pi }$ from $\phi_{z}$, capitalizing on the fact that the original noise-free phase image should be smooth within the fringes. We propose here a modified K-SVD algorithm for interferometric phase denoising, named *Proximity-based K-SVD (ProK-SVD)*.

The conventional K-SVD algorithm has been originally formulated in connection with (digitized) optical image denoising [34]. The semantic and structure of differential interferograms is quite different and hence the performance of K-SVD denoising is not obvious, as discussed below.

### 2.2. The Proximity-Based K–SVD Method

For the sake of the casual reader we summarize here the basics of sparse representations, and the rationale of the conventional K-SVD algorithm. Signal processing techniques for denoising problems require that the chosen representation should efficiently separate signal and noise. Representing a signal translates into the choice of a *dictionary*, a set of elementary signals or *atoms* [21]. Orthogonal dictionaries (bases) have been widely used due to their mathematical simplicity and general applicability. However, orthogonal dictionaries trade generality for compactness of representation. This led to the development of new classes of (overcomplete) dictionaries, which allow to represent specific classes of signal in more compact way. Let us consider the dictionary $D=\left[d_{1}d_{2}\cdots d_{L}\right]\in {ℜ}^{N\times L}$, where the columns constitute the dictionary atoms, and L≥N. Representing a signal $x\in {ℜ}^{N}$, using this dictionary, can follow of two alternative paths: either the *analysis* path, where the signal is represented via its inner products with the atoms,

(5) $$\gamma_{a}=D^{T}x$$

or the *synthesis* path, where it is represented as a linear combination of the atoms,

(6) $$X=D\gamma_{s}$$

In the general case (where the dictionary is not a basis), analysis and synthesis of a signal may differ very much. If D is overcomplete, the family of representations satisfying (6) is actually infinitely large and we can seek the most informative representation of the signal with respect to some cost function C(γ):

(7) $$\gamma_{s}=\underset{\gamma }{\mathrm{argmin}}C\left(\gamma \right)\;\mathrm{Subject}\;\mathrm{to}:\;x=D\gamma$$

In the present context, C(γ) will promote the sparsity of the representation. Then, the dictionary is updated assuming known and fixed coefficients. Given a set of samples $X=\left[x_{1}x_{2},\cdots x_{n}\right]$, the goal of sparse representation is to find a dictionary *D* and a sparse matrix Γ which minimize the representation error,

(8) $$\underset{D,\Gamma }{\mathrm{argmin}}={∥X-D\Gamma ∥}_{F}^{2}\;\mathrm{subject}\;\mathrm{to}:\;{∥\gamma_{i}∥}_{0}\leq T\;\forall i,$$

where $\left\{\gamma_{i}\right\}$ represents the columns of Γ, and the $l^{0}$ sparsity measure ${∥\;\cdot \;∥}_{0}$ counts the number of non-zeros in the representation, and ${∥\;\cdot \;∥}_{F}$ is the Frobenius distance [32,46,47]. The resulting optimization problem is combinatorial and highly non-convex. K-SVD [34] finds a numerical solution to this optimization problem rather than using matrix inversion for dictionary update, changing atom-by-atom via a simple and efficient process.

Applying the original K-SVD algorithm proposed by [34] to differential radar interferogram denoising requires further modification of the algorithm.

**Figure 1:**
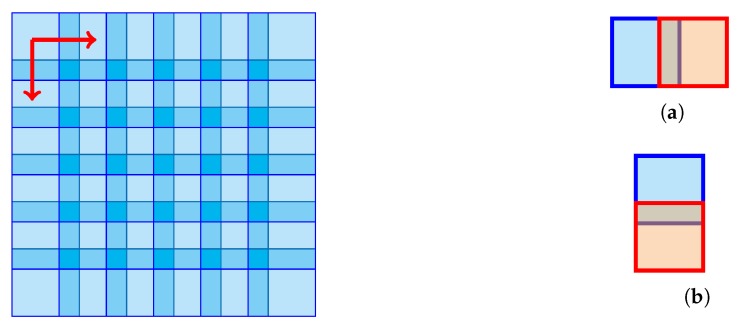
Overlapping patches in Rubinstein K-SVD algorithm and related horizontal (**a**) and vertical (**b**) shift.

**Figure 2:**
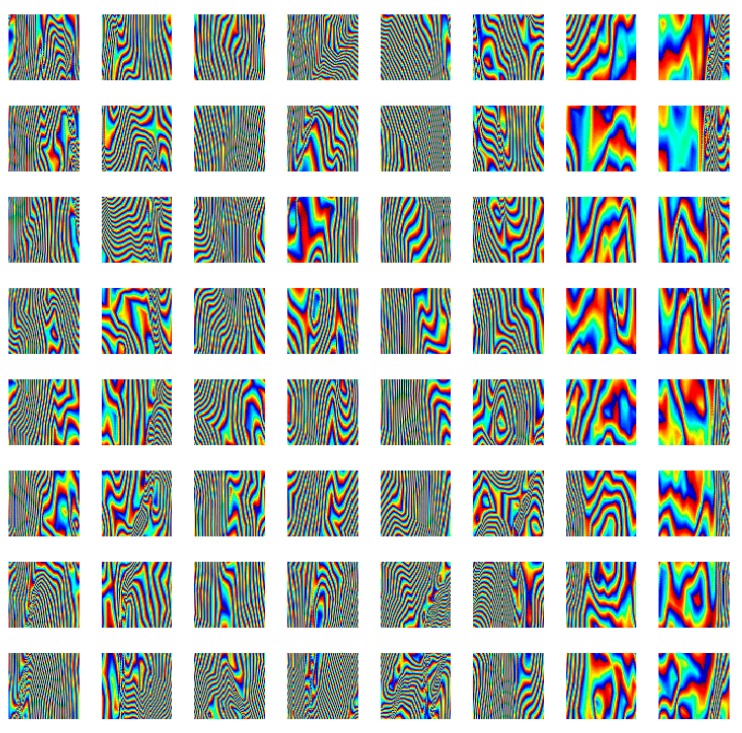
Simulated 512×512 pixels interferogram split into 64 patches, 64×64 pixels each, for PCA analysis.

Following a local approach in [34] and by using the efficient code developed by Rubinstein in [53] modified for our purposes as we shall explain better later, the original two dimensional N×N interferogram is reduced to a one dimensional array whose elements are patches of size n×n, with n≪N to be scanned sequentially, with partial overlap (see Figure 1 ), and *local sparsification* on *each* patch is applied. Mathematically this can be described by

(9) $${\hat{\phi }}_{2\pi }=\left(\sum_{i=1}^{h}\mathrm{argmin}\left[{\hat{\phi }}_{2\pi }\left(i\right)\right]\right)$$

where ${\hat{\phi }}_{2\pi }\left(i\right)$ for i=1,…,h represents the sparsification of the *i*th patch viz.:

(10) $${\hat{\phi }}_{2\pi }\left(i\right)=\hat{\alpha }\left(i\right)\hat{D}\left(i\right)$$

with a dictionary (matrix) of size $D\left(i\right)\in {ℜ}^{n^{2}\;\cdot \;k}$ (with $k\gg n^{2}$), and $\hat{\alpha }\left(i\right)$ defined such that

(11) $$\hat{\alpha }\left(i\right)=\underset{\alpha }{\mathrm{argmin}}{∥\alpha ∥}_{0}\;\mathrm{Subject}\;\mathrm{to}:\;{∥D\left(i\right)\alpha -\phi_{z}\left(i\right)∥}_{2}^{2}\leq \epsilon$$

with $\phi_{z}\left(i\right)$ being the *i*th (noisy) patch. To set the patch dimension *Q* we estimate the number of principal components of the original image. This latter is partitioned into a variable number of patches, and in each patch the number of Principal Component Analysis (henceforth PCA) is computed. Remarkably, irrespective of the partitioning, each patch can be considered as a realization of the stochastic process characterizing the interferogram structure. To estimate the optimal PC-patch dimension we perform an unsupervised training Principal Component Analysis on a simulated interferogram dataset shown in Figure 2.

**Figure 3:**
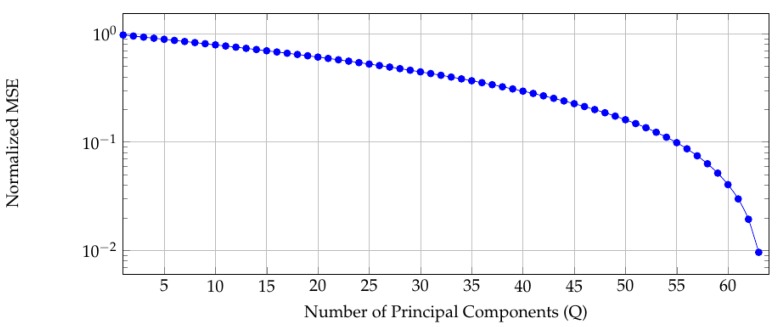
Log-lin plot of normalized mean square error when approximating the noise-free data with *Q* principal components.

The dataset consists of 64 blocks of 4096 elements each. The normalized mean square error between the simulated noise-free data and its patched *Q*-components approximant for varying *Q* are shown in Figure 3. The typical PCA behaviour is observed namely the presence of a knee in the curve for Q≈60. Accordingly, we will use 64 principal components to describe each block.

**Figure 4:**
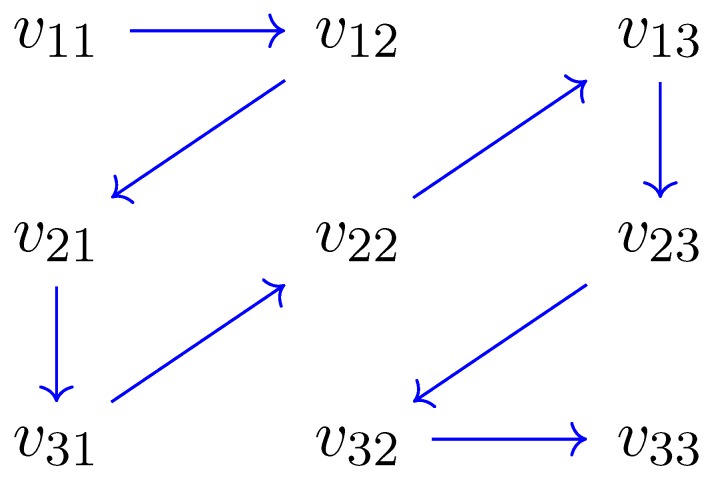
Proximity-based ordering of sample 3×3 patch matrix.

The next step is to reduce each patch to a one dimensional array, as in [53]. However, instead of scanning the patch in columns, appending columns one after another, in order to preserve spatial correlation of the data, we introduce a proximity concept, assuming that proximity implies similarity. Each patch is accordingly scanned as exemplified in Figure 4.

**Figure 5:**
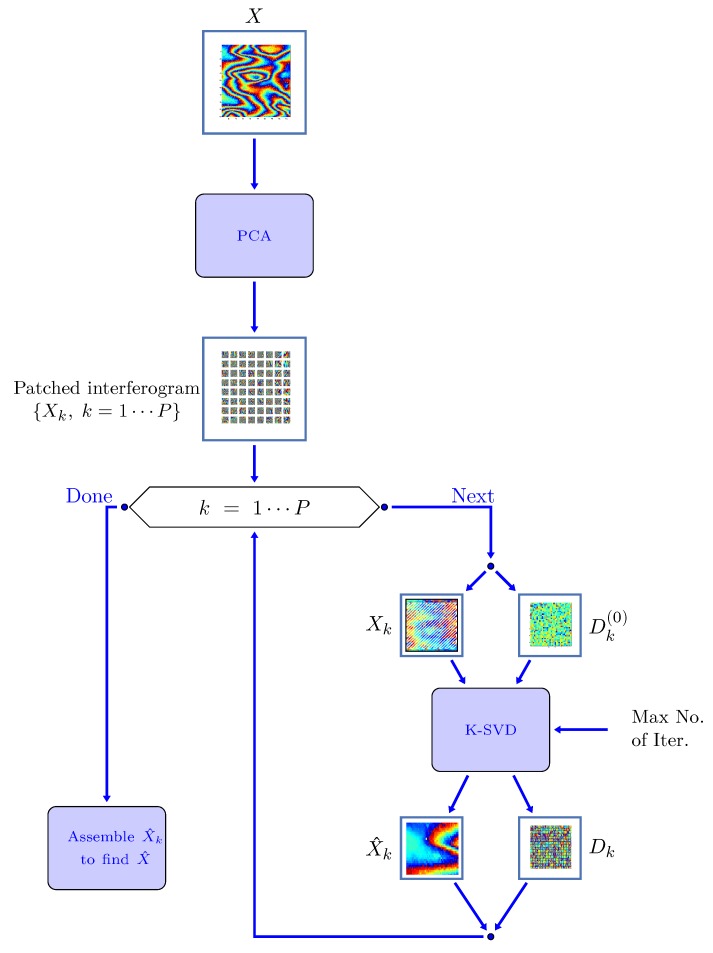
Block diagram of ProK-SVD Synthetic Aperture Radar (SAR) interferogram denoising algorithm. In the first step an unsupervised PCA analysis is implemented aimed at decomposing the original image into *P* patches of size n×n. In the second step each patch $X_{k}$ is re-arranged into a 1D array using proximity, an initial dictionary $D_{k}^{\left(0\right)}$ is chosen, and the K-SVD algorithm is run (up to a suitable maximum number of iteration) to obtain a denoised version of the patch ${\hat{X}}_{k}$, and the related (optimal) dictionary $D_{k}$. Finally, all denoised patches are re-assembled to form the denoised (full) interferogram $\hat{X}$.

We will show that this choice will give better performance than the K-SVD column-by-column scanning. K-SVD is applied to each patch in two steps [54]: (1) a block-coordinate minimization algorithm and (2) a search of optimal ${\hat{\alpha }}_{i}$. Orthonormal matching pursuit [32,54] is used, selecting one atom at a time, and stopping when the error ${∥D\alpha -\phi_{z}\left(i\right)∥}_{2}^{2}$ goes below a fixed threshold. Given all $\hat{\alpha }\left(i\right)$, $\hat{\alpha }$ is updated. The block diagram of the whole processing chain of the proposed ProK-SVD algorithm for SAR interferogram denoising is shown in Figure 5. An initial dictionary is selected to start the process of phase denoising on a single wrapped interferogram from a stack of data. Next the ProK-SVD method is applied in two steps, i.e., sparse representation and dictionary update. When the algorithm meets the preset threshold *T*, the process stops and the denoised phase map is obtained.

In the next section numerical experiments on simulated as well as real data are described and commented.

## 3. Results and Discussion

We discuss here the performance of our ProK-SVD algorithm, using different simulated as well as real interferometric data (provided by CNR-IREA: the ALOS and COSMO-SkyMed data in the frame of the MED-SUV project (http://med-suv.eu/); the ENVISAT and ERS data in the frame of the ASI, DCP and MIUR project ”A multidisciplinary study on the preparatory phases of an earthquake“ (http://www.irea.cnr.it/en/index.php?option=com_k2&view=item&id=545:a-multidisciplinary-study-on-the-preparatory-phases-of-an-earthquake&Itemid=166).).

### 3.1. Simulated Data

**Figure 6:**
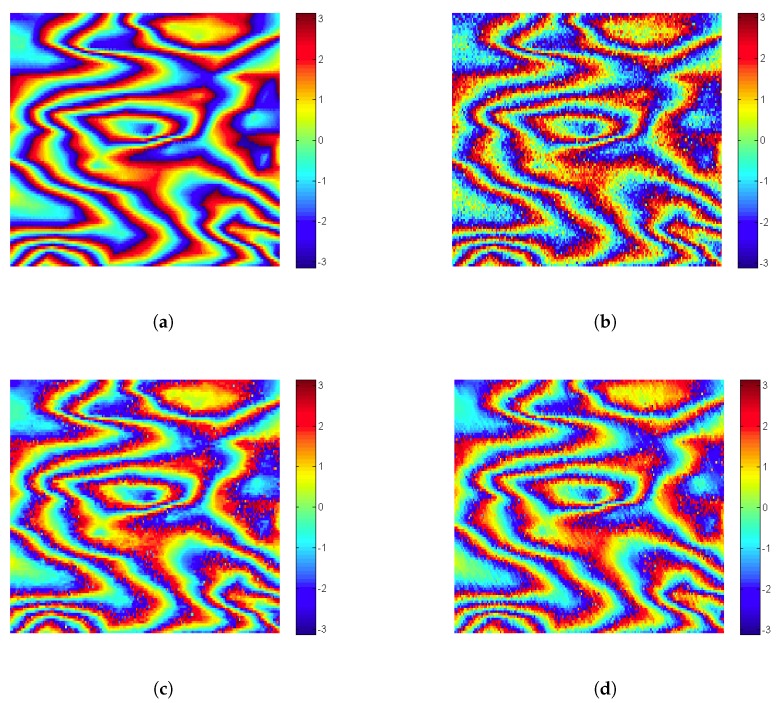
Extracted 100×100 patch of simulated interferogram from ERS data of Rome (Italy): (**a**) simulated noise-free interferogram (*X*); (**b**) simulated data corrupted by an additive white noise with standard deviation equal to 0.5 (Y=X+ν); (**c**) denoised interferogram obtained by K-SVD ($\hat{X}$); (**d**) denoised interferogram obtained by ProK-SVD (${\hat{X}}_{prox}$).

For interferogram simulation, we follow the procedure described in [55], using two SAR acquisitions of the same area, and a Digital Elevation Model (DEM). Specifically, we use a Shuttle Radar Topography Mission (SRTM) DEM of three arcseconds (i.e., 90 m spatial resolution), and two ERS-sensor data of the city of Rome, acquired on December 1st 1996 and on 9 June 1996. In Figure 6a we show a 100×100 close-up of the whole simulated 512×512 interferogram.

In Figure 6b we added zero mean white Gaussian noise with a standard deviation of 0.5 to the simulated fringe pattern. For simplicity we confine our investigation here to additive Gaussian noise, but the proposed method does not rely on any specific assumption about the noise statistics.

For producing Figure 6, the initial dictionaries $D_{k}^{\left(0\right)}$ were chosen as the columns of the $X_{k}$ patch.

**Figure 7:**
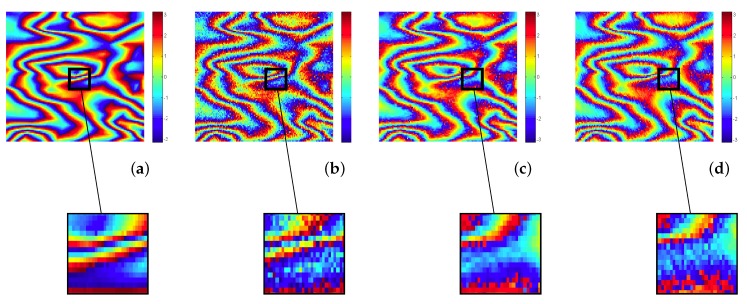
Top: Extracted 100×100 patch from Figure 6: (**a**) simulated fringe pattern (*X*); (**b**) noisy interferogram with $\sigma_{\nu }=0.5$; (**c**) denoised interferogram obtained by K-SVD; (**d**) denoised interferogram obtained by ProK-SVD. Bottom: some close-ups.

Figure 7 shows a close-up of Figure 6 comparing visually the denoising performance of K-SVD and ProK-SVD.

**Figure 8:**
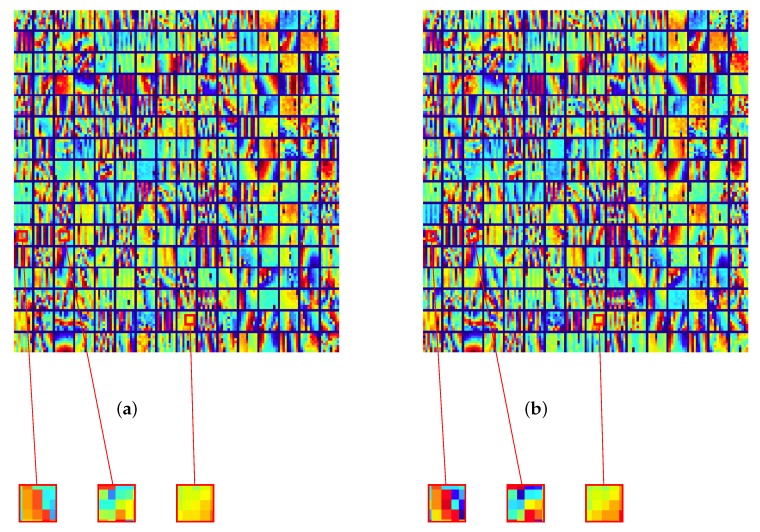
Final update of dictionary, $\left\{D^{\left(k\right)},k=1\cdots ,256\right\}$, for ERS data of Rome (Italy) patched into 16×16 patches, 64×64 pixels each. (**a**) K-SVD and (**b**) ProK-SVD. Some blocks are highlighted to show the effects of proximity.

The final dictionaries $\left\{D_{k}\right\}$ are shown in Figure 8, together with some close-ups whereby the effect of proximity can be qualitatively grasped.

For each (n×n) patch we may estimate the local Peak Signal-to-Noise Ratio ($PSNR_{k}$) [56]

(12) $$PSNR_{k}=20\;\cdot \;\log_{10}\left[\frac{max(X_{k})}{\sqrt{MSE_{k}}}\right]\mathrm{where}\;\;MSE_{k}=\frac{1}{n^{2}}\sum {\left\|X_{k}-{\hat{X}}_{k}\right\|}^{2}$$

**Figure 9:**
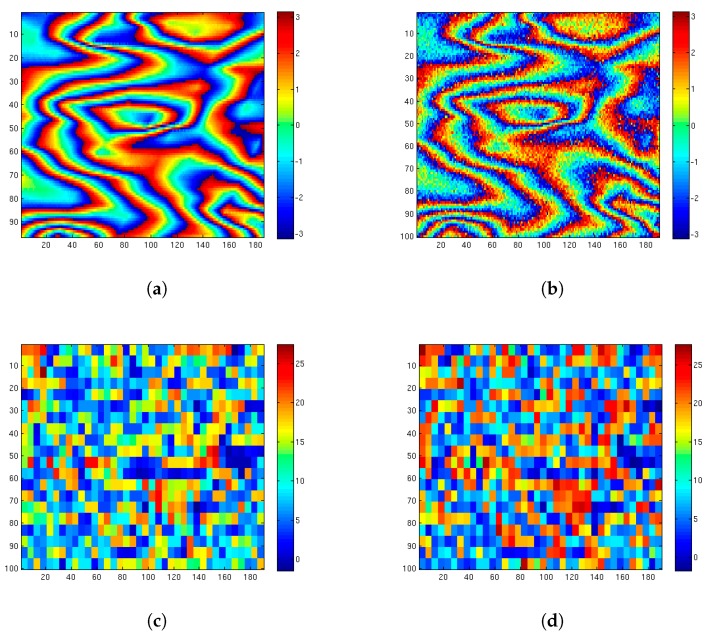
Extracted 100×190 patch of a simulated interferogram: (**a**) simulated fringe pattern; (**b**) simulated data corrupted by an additive white noise with standard deviation equal to 0.5; (**c**) Peak Signal-to-Noise Ratio (PSNR) map for K-SVD; (d) PSNR map for ProK-SVD.

$X_{k}$ beeing the true and ${\hat{X}}_{k}$ the estimated phase. The map of the (local) $PSNR_{k}$ for K-SVD and ProK-SVD is displayed in Figure 9 for the simulated interferogram shown in Figure 6.

**Figure 10:**
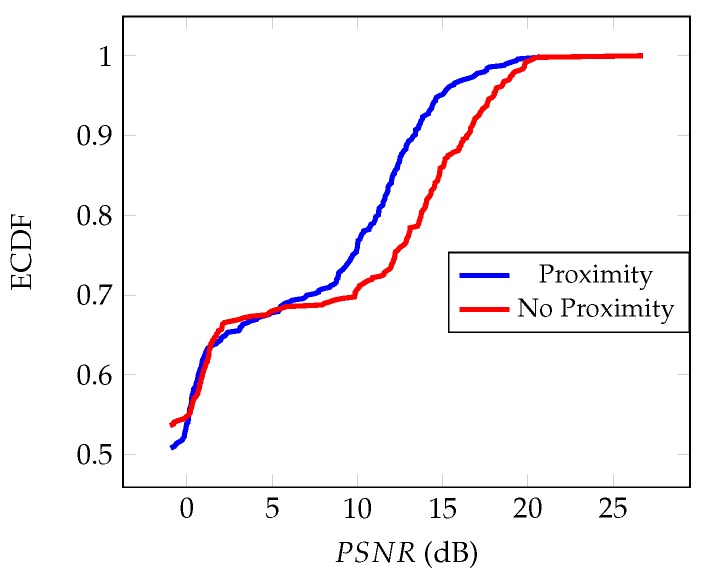
Empirical Cumulative Distribution Function of PSNR, Equation (12), for K-SVD denoised simulated interferogram with and without proximity.

The related (empirical) distribution functions of the $PSNR_{k}$ for the K-SVD and ProK-SVD denoised interferograms are shown in Figure 10. It is seen that proximity boosts the performance of K-SVD in the range [5–10] dB.

The average of the quantities (12) over all patches making up our test-image is given in Table 1.

Overall, the proximity strategy helps extracting the structure of the original phase map in a more faithful way. Accordingly, we will adopt the ProK-SVD in our further experiments on real data.

Different choices of the initial dictionaries $D_{k}^{\left(0\right)}$ are obviously possible. Specifically, we consider two additional possible choices: an Overcomplete Discrete Cosine Transform (ODCT) dictionary, and a Random Dictionary (RD).

The ODCT dictionary has a strong *energy compaction* property, tending to concentrate the signal features in a few low-frequency components. The RD taken from a uniform distribution in the interval $\left.\left[-\pi ,\pi \right]\right.$ provides a structure-free playground. The ODCT and RD do not depend on the content and noise level of the image. The DD dictionary on the other hand is built from the noise corrupted interferogram.

**Figure 11:**
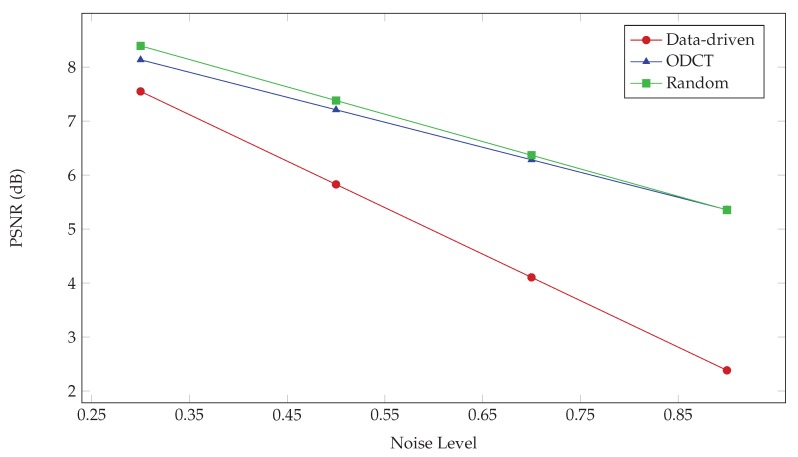
Plot of PSNR (dB) vs. noise level (ν) for simulated data using three types of training dictionaries in ProK-SVD.

The performance of the above dictionaries for different noise levels is compared in Figure 11 for the considered simulated interferogram in terms of the average (over patches) PSNR. It is seen that the ODCT and RD behave almost equivalently better than the DD dictionary.

**Figure 12:**
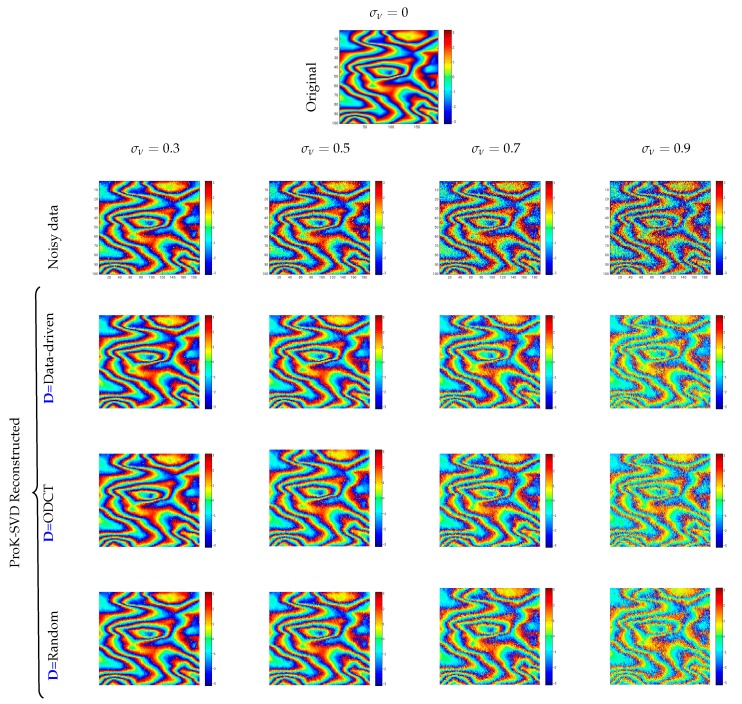
On top the original noisy-free map is shown. The second row displays the original phase map corrupted by additive noise with given standard deviation. The subsequent rows describe the reconstruction obtained using ProK-SVD for different initial dictionary **D**.

A visual comparison of the denoised interferograms obtained using the DD, ODCT, and RD initial dictionaries, for various added noise levels, is shown in Figure 12. It is visually evident that for low to moderate noise levels the random dictionary provides the best (smoothest, more faithful) reconstruction. For any fixed dictionary the reconstruction becomes worse as the noise level is increased, in particular loosing contrast.

**Figure 13:**
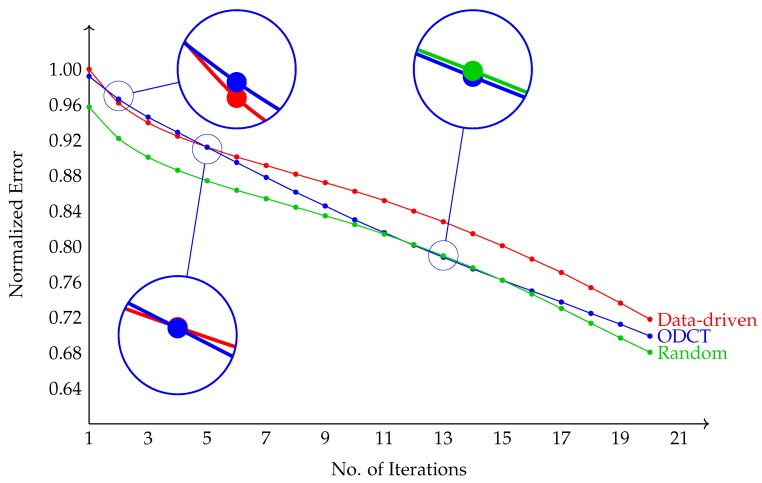
Iterative algorithm convergence: $l^{2}$ norm of the Γ matrix coefficients (see Equation (8)) versus number of iterations for three different chosen dictionaries: DD (blue), ODCT (red), RD (green).

The choice of the dictionary has little impact on the number of iterations required in the K-SVD step of the algorithm, as illustrated in Figure 13, in terms of the $l^{2}$ reconstruction error in Equation (12) versus the number of iterations.

### 3.2. Real Data

On the basis of the above, we finally illustrate the performance of ProK-SVD on real SAR interferometric data, using the RD initial dictionary.

On purpose we use interferometric data pairs from various SAR platforms over the area of the Etna Volcano (Italy), namely archived data from the ERS, ENVISAT, ALOS, and COSMO-SkyMed SAR sensors, with varying spatial and temporal baselines summarized in Table 2. Data having smaller spatio-temporal baseline, e.g., in our case COSMO-SkyMed and ENVISAT have lower interferometric noise. Conversely data having large spatial temporal baselines, i.e., ALOS and ERS have higher noise.

**Figure 14:**
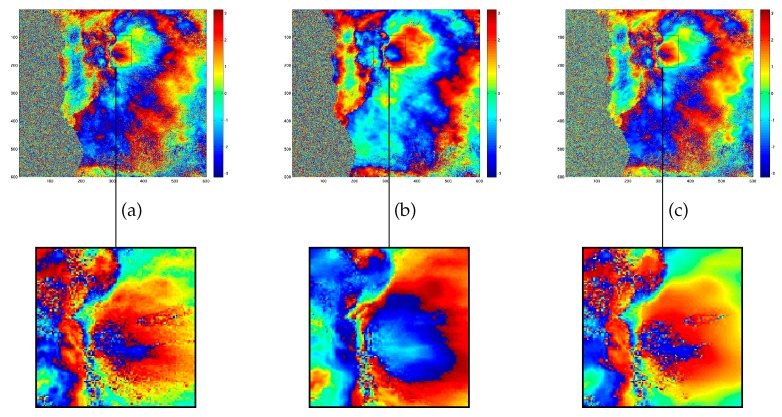
Comparison among different filtering algorithms. Extracted 700×700 patch of interferogram of ENVISAT SAR sensor over the Etna Volcano area. Top row: (**a**) original interferogram; (**b**) Goldstein Filtering with filter parameter value equal to 0.5; (**c**) ProK-SVD. Bottom row close-ups.

As a first benchmark we consider Goldstein filtering [7], widely used in the conventional SAR denoising techniques, with the power factor α=0.5 (As shown in Figure 4 of Baran et al. (2003), the performance of the plain and modified Goldstein algorithm are comparable in the coherence range [0.4, 0.8] for the chosen filter parameter α=0.5), to denoise an ENVISAT interferogram over the Etna Volcano. In Figure 14 the ProK-SVD results are visually compared to those obtained from Goldstein technique.

Assessing the quality of image denoising algorithms in the case where no noise-free version is available is an extensively studied (and still open) issue. Several quality metrics have been proposed in the literature, as discussed e.g., in [57]. We adopt the signal-to-distorsion ratio (SDR), discussed, e.g., in [58], defined as

(13) $$SDR=10\;log10\left[\frac{\sum_{i,j}Y{\left(i,j\right)}^{2}}{\sum_{i,j}{\left(Y\left(i,j\right)-\hat{X}\left(i,j\right)\right)}^{2}}\right]$$

that represents the ratio of the energies of the noisy phase map and the energy of the (fiducial) noise removed by the de-noising process.

**Figure 15:**
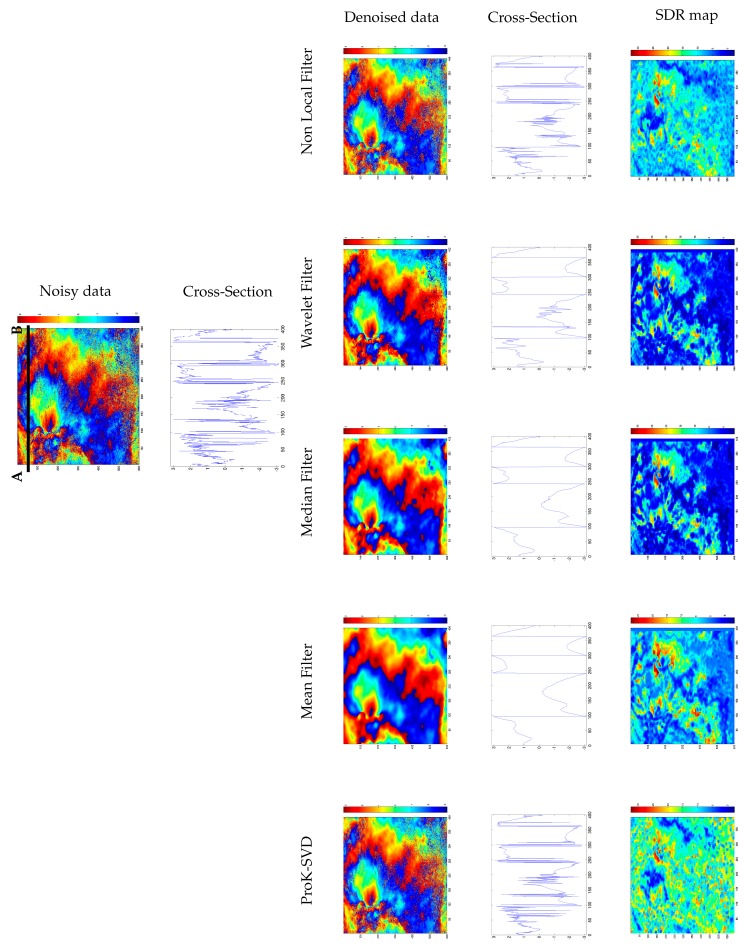
Comparison among ProK-SVD and several denoising techniques: at the top extracted 600×400 patch of the noisy interferogram with a phase cross section profile relevant to the A-B section. The third row shows the results of the denoised process using ProK-SVD, Mean filter, Median Filter, Wavelet Filter, and Non-Local filter. The fouth row displays the corresponding phase cross section profiles. The last row presents the Signal-to-Distortion Ratio (SDR) maps for each technique.

In Figure 15 we extend the comparison to other denoising techniques (in order to preserve phase circularity, the mean, median, and wavelet filters have been applied to the complex interferogram and then the wrapped filtered phase has been extracted). It is noted that the ProK-SVD entails minimum smoothing, and yields a higher SDR while producing effective denoising.

In Table 3 the various denoising techniques are accordingly compared in terms of the average SDR.

**Figure 16:**
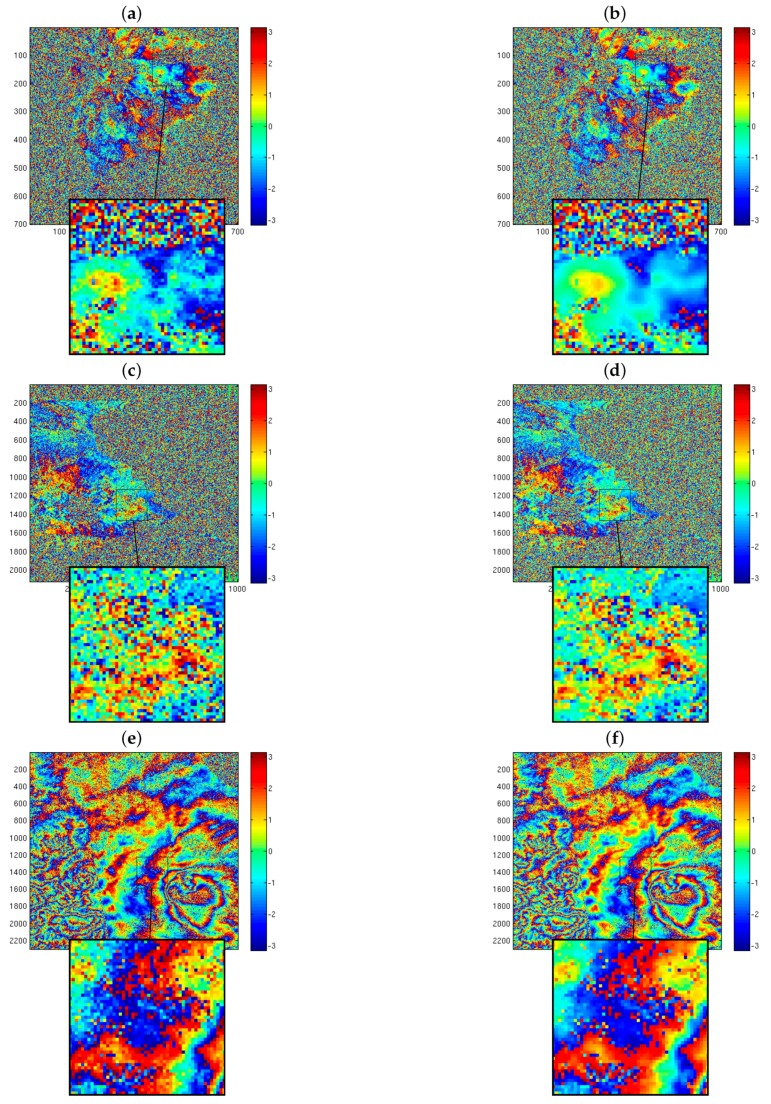
ProK-SVD denoised interferograms of several SAR sensors over the area of the Etna Volcano (Italy). (**a**) ERS original interferogram, and zoomed view; (**b**) ERS denoised interferogram, and zoomed view; (**c**) ALOS original interferogram, and zoomed view; (**d**) ALOS denoised interferogram, and zoomed view; (**e**) COSMO-SkyMed original interferogram, and zoomed view; (**f**) COSMO-SkyMed denoised interferogram, and zoomed view.

Figure 16 show the retrieved interferograms for ERS, ALOS, and COSMO-SkyMed data, with a rectangular box marking a zoomed-in view of the area.

All zoomed views display significant reduction of the noise level compared to the original data. The ProK-SVD technique is seen to preserve effectively the local features within the fringe pattern without any sensible loss of valuable information, and without introducing any artifacts.

This is further illustrated by the values of SDR collected in Table 4.

Summing up, the ProK-SVD approach performs well for all SAR sensors considered, in terms of noise suppression and lack of artifacts.

## 4. Conclusions

In this paper we addressed the interferometric phase image denoising problem by solving the sparse and redundant representations problem over a trained dictionary. The key new idea is to apply a proximity-based modified K-SVD algorithm to the noisy interferograms so as to obtain both a sparse representation and an updated dictionary together. This approach, remarkably, does not require any fine tuning of the relevant parameters, nor any a priori information to work reliably. We tested the proposed algorithm on both simulated and real SAR interferometric data, from low resolution (ERS and ENVISAT), medium resolution (ALOS), and high resolution (COSMO-SkyMed) data. We discussed the choice of the initial dictionaries, referring to three particular cases, namely, random, ODCT and data-driven, and evaluated their performances on simulated data with additive (Gaussian) noise with varying sigma values. The random dictionary was found to yield the best performance. The proposed technique was capable of effectively retrieving the fringe pattern from the noise interferograms, without introducing significant artifacts in a wide range of signal to noise ratios.

As far as computational complexity and burden are concerned, assuming a dictionary of dimension N×L and *T* iterations (see Equation (8)), the ProK-SVD algorithm is dominated by the K-SVD stage, requiring $T^{2}L+2NL$ floating point operations, as demonstrated in [53]. We plan to implement the algorithm using parallel (GPU) architectures for further optimization. 

## Figures and Tables

**Table 1:** Peak Signal-to-Noise Ratio (PSNR) and Mean Standard Error (MSE) metrics for noisy simulated interferogram [ν=0.5], denoised by K-SVD and ProK-SVD.

Noisy Interferogram	Denoised Interferogram	Denoised Interferogram
	from K-SVD	from ProK-SVD
	$\hat{X}$	${\hat{X}}_{prox}$
Y=X+ν	**PSNR(dB)**	
	10.5138	11.5843
	**MSE(dB)**	
	−2.6650	−3.7355

**Table 2:** Synthetic Aperture Radar (SAR) interferometric data pairs of the Etna Volcano used for validation of ProK-SVD.

SENSORS	COSMO-SkyMed	ALOS	ENVISAT	ERS
**Band**	X	L	C	C
**Spatial resolution [m]**	3	10	30	30
**1st acquisition [d/m/y]**	25/11/2009	30/01/2008	15/09/2004	24/11/2004
**2nd acquisition [d/m/y]**	04/12/2009	01/05/2008	20/10/2004	07/06/2006
**Perpendicular baseline [m]**	49.9241	840.309	−19.0227	−197.872
**Time interval [days]**	10	122	35	545

**Table 3:** Signal-to-Distortion Ratio (SDR) values relevant to ProK-SVD, K-SVD, Goldstein filter with parameter α=0.5, non-local filter, wavelet filter, median filter, mean filter (ENVISAT SAR sensor data-set).

ProK-SVD	KSVD	Goldstein	Non-Local	Wavelet	Median	Mean
SDR [dB]						
13.0450	12.9350	−2.8505	9.6538	3.9728	3.7970	3.4605

**Table 4:** SDR of retrieved SAR interferometric data-sets.

Serial Nr.	Study Area	Data	SDR [dB]
1	Etna Volcano	COSMO-SkyMed	13.0617
2		ALOS	15.1970
3		ERS	15.1383
